# Chediak-Higashi Syndrome With Epstein-Barr Virus Triggered Hemophagocytic Lymphohistiocytosis: A Case Report

**DOI:** 10.7759/cureus.11467

**Published:** 2020-11-13

**Authors:** Nishant Gopaal, Jagdish N Sharma, Vijay Agrawal, Sawai S Lora, Laxman S Jadoun

**Affiliations:** 1 Pediatric Medicine, Swai Man Singh Medical College, Jaipur, IND

**Keywords:** chediak-higashi syndrome, silver hair, hemophagocytic lymphohistiocytosis, azurophilic granules, hair microscopy, epstein-barr virus

## Abstract

Chediak-Higashi syndrome (CHS) is a rare, autosomal-recessive disorder characterized by oculocutaneous albinism, recurrent bacterial infections, progressive neurologic abnormalities, coagulation defects and a high risk of developing hemophagocytic lymphohistiocytosis characterized by pancytopenia, high fever, and lymphohistiocytic infiltration of liver, spleen, and lymph nodes. Treatment of accelerated-phase CHS is difficult with poor prognosis. Here, we report a two-and-a-half-year-old male child who was diagnosed with Chediak-Higashi Syndrome based on silvery hair, pathognomonic hair microscopy and giant azurophilic granules in granulocytes. The patient was in advanced stage of HLH induced by an Epstein-Barr virus (EBV) infection and given etoposide, cyclosporine and dexamethasone according to hemophagocytic lymphohistiocytosis (HLH)-2004 protocol but did not survive.

## Introduction

Chediak-Higashi syndrome (CHS) is a rare, autosomal-recessive disorder with less than 500 cases published worldwide in the last 20 years [1]. It is caused by a defect in the lysosomal trafficking regulator gene CHS1/LYST which is part of a family of vesicle trafficking regulatory proteins.

It is characterized by silvery hair, recurrent bacterial infections, progressive neurologic abnormalities and coagulation defects. Approximately 85% of the cases develop an accelerated phase characterized by pancytopenia, hemophagocytosis, and marked infiltration of organs by lymphocytes, leading to multi-organ dysfunction [2].

The diagnosis of CHS is suggested by characteristic findings on hair microscopy and pathognomonic giant cytoplasmic granules in leukocytes on a peripheral smear. Confirmation is made by the identification of a pathogenic variant in the CHS1/LYST gene. Allogeneic hematopoietic cell transplantation (HCT) is the treatment of choice [1].

## Case presentation

**Figure 1:**
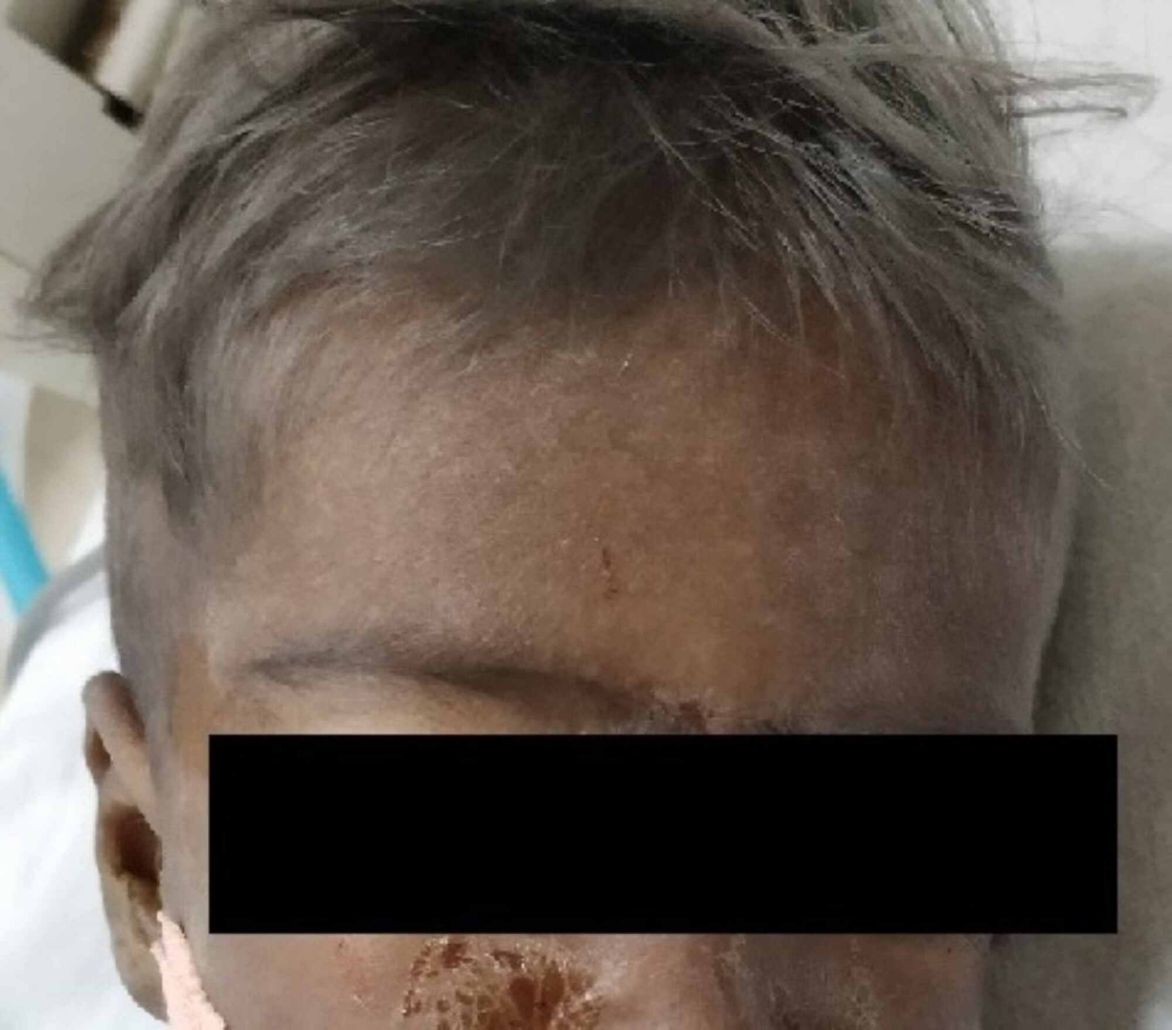
Silvery-grey hair

A 30-month-old male child born of consanguineous marriage presented with a history of seizures, fever, abdominal distention, yellowish discoloration of eyes and urine for one month. Birth, developmental and family history were unremarkable. On physical examination, the child had silvery grey hair, hypopigmented skin, pallor, icterus, splenomegaly (span-12 cm), hepatomegaly (10 cm), cervical and axillary lymphadenopathy (Figure 1). The patient had moderate respiratory distress with bilateral coarse crepitations. Patient was hypotensive (60/40 mmHg) and developed seizures soon after admission and was promptly started on intravenous levetiracetam and vasopressors. The patient soon developed respiratory failure and was taken on ventilator support.

CBC showed pancytopenia (TLC-9,700/mm^3^ - 60% neutrophilic; Hb-5.9 g/dL; platelet count- 20,000/mm^3^). LFT revealed direct hyperbilirubinemia (total Bilirubin- 10.16; direct-6.94; indirect-3.22), mildly raised Transaminases (AST-103 IU/L; ALT-88 IU/L ), deranged coagulation profile (PT 30.4 sec; INR 2.40); total protein (4.36g/dL); LDH (826 U/L), GGT (45 U/L), hypertriglyceridemia (319 mg/dL), raised ferritin (3029 ng/mL); USG abdomen showed hepato-splenomegaly with normal echotexture. X-ray showed bilateral lung infiltrates. The child was treated empirically with ceftriaxone, vancomycin, and fluconazole.

**Figure 2:**
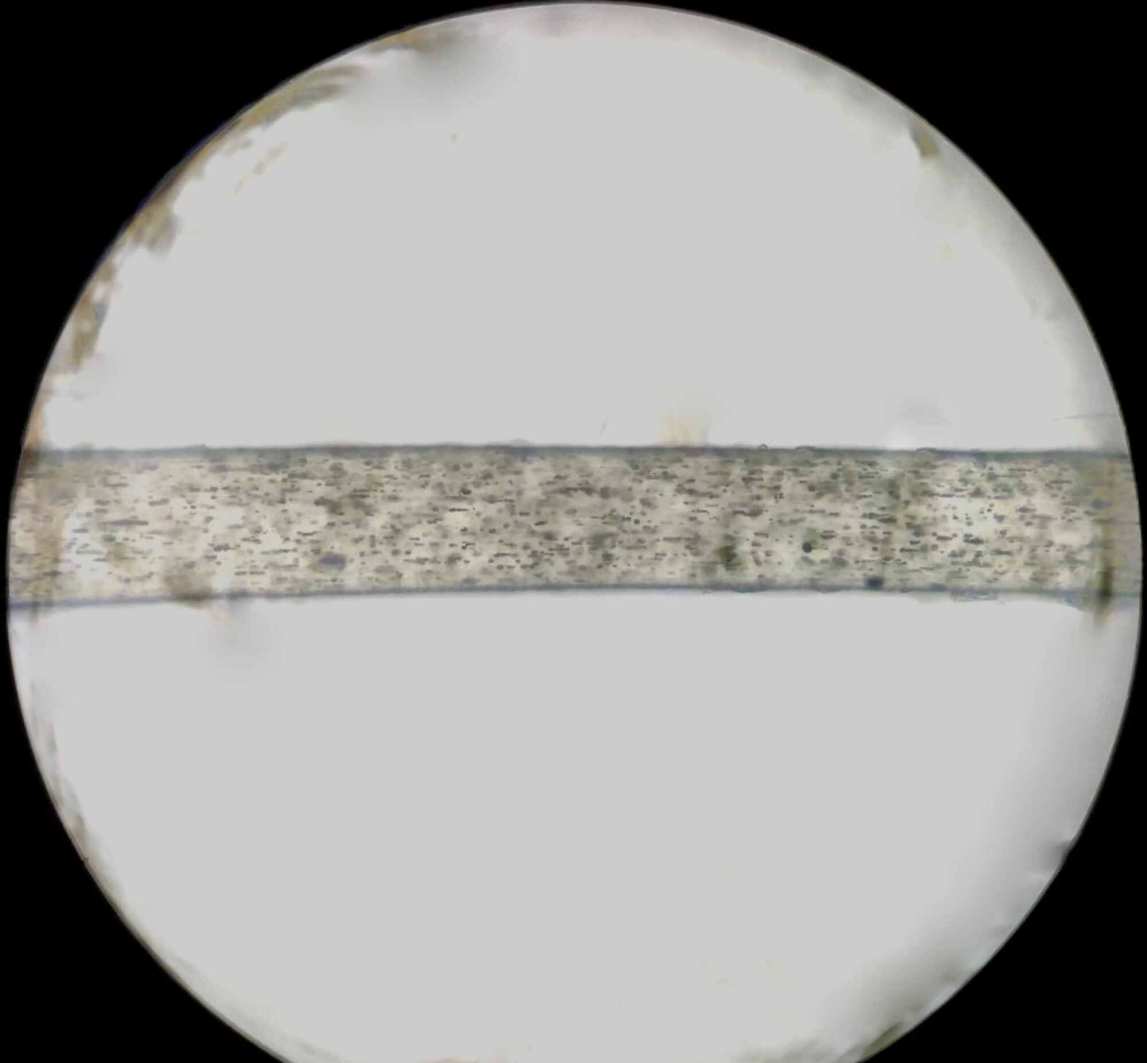
Hair microscopy of the silvery grey hairs showing evenly distributed melanin granules of regular diameter along the shaft

**Figure 3:**
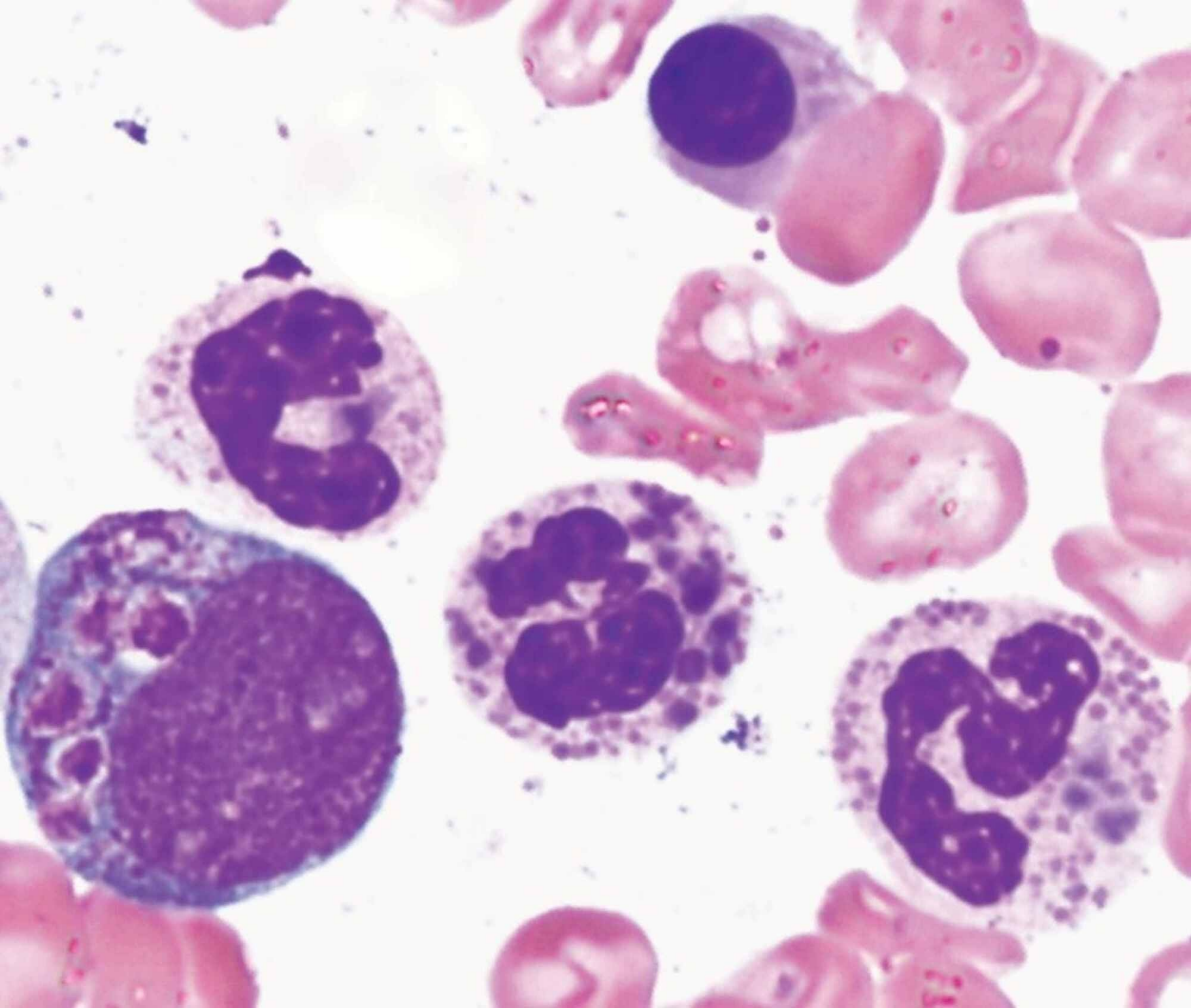
Peripheral blood smear showing classic giant azurophilic peroxidase-positive granules in neutrophils

In view of these findings, a differential diagnosis of CHS, Griscelli syndrome and Hermansky-Pudlak syndrome was made. Hair microscopy of the silvery grey hairs showed evenly distributed melanin granules of regular diameter along the shaft (Figure 2). Peripheral blood smear revealed the classic giant azurophilic peroxidase-positive granules in neutrophils, typically seen in CHS (Figure 3). Thus, a final diagnosis of CHS was made based on the characteristic findings of PBF and hair microscopy.

Five out of the eight diagnostic criteria for hemophagocytic lymphohistiocytosis (HLH) were fulfilled (fever, splenomegaly, pancytopenia, raised ferritin and hypertriglyceridemia). A cause for the accelerated phase was sought and blood culture, urine culture, Chest X-ray and viral markers were sent. The patient tested positive for IgM antibodies to the Epstein-Barr virus (EBV) by enzyme-linked immunosorbent assay (ELISA).

In view of deteriorating organ function patient was promptly started on intravenous dexamethasone, cyclosporin and etoposide for management of HLH. Multiple packed cell and FFP were transfused. The patient expired on day 4 of hospitalization due to multi-organ dysfunction.

## Discussion

CHS is a rare autosomal-recessive disorder with an estimated incidence of <1 in 1,000,000 and fewer than 500 cases reported worldwide. Mean age of onset is six years and most do not survive beyond 10 years. It is caused by mutations in the CHS1/LYST gene at 1q42.1-2 locus that control vesicle trafficking regulatory proteins and leads to aberrant fusion of vesicles and failure to transport lysosomes to the appropriate site of action. It is characterized by severe immunodeficiency, hypomelanosis, silvery grey hair, neurologic abnormalities, coagulation defects, and a high risk of developing hemophagocytic lymphohistiocytosis [1].

Hypopigmentation of the skin and hair is caused by the presence of large aggregates of pigment in hair shafts and the accumulation of mature melanosomes in melanocytes [3]. Neutrophils have giant azurophilic granules which do not appropriately release their contents in the setting of bacterial or viral infections and lead to impaired bactericidal and cytotoxic function of NK cells and T-cells. Patients consequently develop recurrent skin and respiratory infections most commonly due to Staphylococcus aureus and beta-hemolytic Streptococcus [4].

The constellation of symptoms of silvery grey hairs, hepatosplenomegaly, neurological symptoms and coagulation defect raised the possibility of CHS, Griscelli syndrome as possible diagnosis. In CHS hair microscopy characteristically shows evenly distributed melanin granules of regular diameter, while in Griscelli Syndrome hair shafts present with large uneven melanin granules mostly located in the vicinity of the medullar zone [5]. Classic giant azurophilic granules are seen in all granule-containing cells, including peripheral blood and bone marrow cells, melanocytes, peripheral and central nerve tissue in CHS but are absent in Griscelli syndrome [6].

Definitive diagnosis is based on genetic testing for mutations in the CHS1/LYST gene. Most mutations are unique. Thus, identifying the exact mutation and differentiating true disease-causing mutations from benign genetic variants can be a challenge. Prenatal diagnosis is also possible and is commonly performed by capillary sequencing of the CHS1/LYST gene [7].

Cytotoxic T-cell and NK cell dysfunction predispose to the development of HLH, especially with viral infections. In most of the virus associated cases, herpes group viruses (i.e., EBV, cytomegalovirus, herpes simplex virus and varicella zoster) have been identified as the presumed etiologic agents [7].

Despite increasing evidence that hemophagocytic syndrome is virus-associated in the CHS patients, possibility of nonviral stimuli or underlying immunoregulatory abnormalities alone triggering the syndrome cannot be ruled out. CHS patients are predisposed to malignancy due to a defect in their DNA repair ability and to spontaneously lyse various tumour cells [8]. Prospective studies are needed to monitor patients with CHS for intercurrent viral infections and to correlate such an infection with the development of HLH.

Guidelines for diagnosis and treatment of HLH were developed by HLH-2004 group [9]. Five of the eight criteria must be fulfilled, but patients with a molecular diagnosis consistent with HLH do not necessarily need to fulfil the diagnostic criteria. The criteria include (1) fever, (2) splenomegaly, (3) cytopenias affecting at least two of three lineages in the peripheral blood, (4) hypertriglyceridemia and/or hypofibrinogenemia, (5) hemophagocytosis in bone marrow, spleen, or lymph nodes, (6) low or absent NK-cell activity, (7) hyperferritinemia, and (8) high levels of sIL-2r.

Treatment protocol according to HLH-2004 group is designed for the patients with HLH, with or without evidence of familial or genetic disease, regardless of suspected or documented viral infections. Horne et al. demonstrated that patients with EBV infection and a clinical picture of HLH have a significant advantage when treated according to this protocol [10]. Combination therapy consists of etoposide, dexamethasone, and cyclosporine A. remission is achieved in 75% of individuals within eight weeks; however, relapses are common and response to treatment declines over time. Once remission occurs, prompt HSCT is recommended. HLH and its complications are the most common cause of mortality in individuals with CHS and the prognosis is poor once HLH phase sets in [11].

## Conclusions

In conclusion, CHS is a rare disease with similar clinical spectrum as, Griscelli syndrome, Hermansky-Pudlak syndrome and other immunodeficiency syndromes. The prognosis of the HLH phase is poor and hence early diagnosis on the basis of characteristic clinical findings and diagnostic laboratory examinations is critical to facilitate timely bone marrow transplantation before the development of accelerated phase.
